# Single and Multi-trait GWAS Identify Genetic Factors Associated with Production Traits in Common Bean Under Abiotic Stress Environments

**DOI:** 10.1534/g3.119.400072

**Published:** 2019-05-27

**Authors:** Atena Oladzad, Timothy Porch, Juan Carlos Rosas, Samira Mafi Moghaddam, James Beaver, Steve E. Beebe, Jimmy Burridge, Celestina Nhagupana Jochua, Magalhaes Amade Miguel, Phillip N. Miklas, Bodo Ratz, Jeffery W. White, Jonathan Lynch, Phillip E. McClean

**Affiliations:** * Department of Plant Sciences, North Dakota State University, Fargo, ND, 58102; † USDA-ARS, Tropical Agricultural Research Station Mayaguez Puerto Rico; ‡ Department of Agricultural Engineering, Zamorano University, Zamorano, Honduras; § Plant Resilience Institute, Department of Plant Biology, Michigan State University, East Lansing, MI, 48824; ** Department of Agronomy and Soils, University of Puerto Rico, Mayaguez, Puerto Rico 00680; †† International Center for Tropical Agriculture (CIAT), Cali, Colombia; ‡‡ Department of Plant Science, Pennsylvania State University, State Collage, PA, 16801; §§ South Zonal Center, Maputo, Mozambique; *** Mozambic Institute of Agricultural Research, Chimoio, Mozambic; ††† USDA-ARS, Grain Legume Genetics Physiology Research, Prosser, WA; †† International Center for Tropical Agriculture (CIAT), Cali, Colombia; ‡‡‡ USDA-ARS, Plant Physiology and Genetics Research Maricopa, AZ

**Keywords:** *Phaseolus vulgaris*, gene pool, Middle American, Andean, heat stress, drought stress, haplotype map

## Abstract

The genetic improvement of economically important production traits of dry bean (*Phaseolus vulgaris* L.), for geographic regions where production is threatened by drought and high temperature stress, is challenging because of the complex genetic nature of these traits. Large scale SNP data sets for the two major gene pools of bean, Andean and Middle American, were developed by mapping multiple pools of genotype-by-sequencing reads and identifying over 200k SNPs for each gene pool against the most recent assembly of the *P. vulgaris* genome sequence. Moderately sized **B**ean **A**biotic **S**tress **E**valuation (BASE) panels, consisting of genotypes appropriate for production in Central America and Africa, were assembled. Phylogenetic analyses demonstrated the BASE populations represented broad genetic diversity for the appropriate races within the two gene pools. Joint mixed linear model genome-wide association studies with data from multiple locations discovered genetic factors associated with four production traits in both heat and drought stress environments using the BASE panels. Pleiotropic genetic factors were discovered using a multi-trait mixed model analysis. SNPs within or near candidate genes associated with hormone signaling, epigenetic regulation, and ROS detoxification under stress conditions were identified and can be used as genetic markers in dry bean breeding programs.

Common bean (*Phaseolus vulgaris* L.) is the most important and affordable food legume for over 80 million poor people in regions of Latin America, the Caribbean, and Eastern and Southern Africa. Major consumers of common bean in these countries often live on less than two U.S. dollars per day, where beans are grown primarily by smallholder farmers on less than two hectares (McClean and Raatz 2017). Bean productivity on these farms is reduced by high ambient temperatures and drought that affect development and reproduction (Buruchara *et al.* 2011). In East Africa, 70% of bean production is threated annually by drought and high night-time temperatures. Therefore, there is a real need to improve common bean productivity in these regions for fast growing populations that will be affected by anticipating future conditions brought on by climate change (Beebe *et al.* 2011). Improvement efforts are best supported by genetic analyses of important production traits since stable genetic changes have a longer lasting effect on productivity (Mukankusi *et al.* 2018). Recent advancements such as a reference bean genome sequence (Schmutz *et al.* 2014), genotype-by-sequencing methods (GBS; Schröder *et al.* 2016), GWAS (Moghaddam *et al.* 2016), and available database resources (http://phaseolusgenes.bioinformatics.ucdavis.edu/) are enabling the discovery of genetic factors associated with the abiotic stress response. The first dense genotyping tool was the 6k Illumina Infinium SNP assay (Song *et al.* 2015). This platform proved useful for the discovery of many important agronomic traits primarily with bi-parental mapping studies of common bean (Mukeshimana *et al.* 2014; Brisco *et al.* 2014; Bello *et al.* 2014). However, recent GBS methods generated a much higher number of SNPs per population for fine-mapping genomic regions of interest (Moghaddam *et al.* 2016).

A genetic discovery population carefully designed to include variation for response to heat and/or drought stress is important for discovering critical genetic factors associated with the abiotic stress response. Initial abiotic stress tolerance studies in common bean used bi-parental populations (Blair *et al.* 2012; Mukeshimana *et al.* 2014; Villordo-Pineda *et al.* 2015) and discovered several quantitative trait loci (QTL) for each agronomic trait evaluated under drought and/or heat stress. Bi-parental population studies are important to discover rare alleles with large effects (Singh and Singh 2015). By contrast, association panels can sample variation across a larger number of genotypes and be used to discover both large or small effect genetic factors that are associated with the plant’s response to abiotic stress conditions (Risch 2000; Mamidi *et al.* 2011b; Moghaddam *et al.* 2016).

Wild Middle American (MA) and Andean common bean gene pools evolved from an ancestral population ∼110,000 years ago (Mamidi *et al.* 2013), and independent domestication events occurred in the two wild pools ∼7,000 years ago (Mamidi *et al.* 2011a). Repeated studies have shown genetic diversity is greater among domesticated MA beans than domesticated Andean beans (Velasquez and Gepts 1994; Mamidi *et al.* 2013; Schmutz *et al.* 2014). Each gene pool has specific agronomic, morphological, physiological and molecular characteristics, and allele frequencies differ between the two gene pools for genetic factors controlling a trait (Singh *et al.* 1991; Schmutz *et al.* 2014; Lobaton *et al*; 2018). When designing populations for global projects it is important to consider the fact that beans produced in North America, Central American, and parts of South America are typically members of the MA gene pool, while much of the bean production in the remainder of the world focuses on Andean beans. For example, a MA diversity panel (MDP; n∼300) was developed for the USDA funded BeanCAP project that consisted of germplasm grown in the major US production regions from the 1930s to the 2000s (Moghaddam *et al.* 2016). That population was used to identify candidate genes for production (Moghaddam *et al.* 2016), nutritional (Mafi Moghaddam *et al.* 2017; McClean *et al.* 2017), and domestication traits such as increased leaf and seed size (Schmutz *et al.* 2014). Recently, an Andean Diversity Panel (ADP; n∼350) was developed (Cichy *et al.* 2015a), and used to map traits associated with for cooking time (Cichy *et al.* 2015b), disease resistance (Zuiderveen *et al.* 2016, Tock *et al.* 2017), symbiotic nitrogen fixation (Kamfwa *et al.* 2015a), flooding tolerance (Soltani *et al.*, 2018), and agronomic traits such as phenology, aboveground biomass, and seed yield (Kamfwa *et al.* 2015b). The USAID Climate Resilience Bean project (CRIB; https://plantscience.psu.edu/research/labs/roots/projects/usaid-crb) was initiated to understand the genetics and physiological mechanisms of the response of dry beans under abiotic stress environments. One component of the project was to develop appropriate sized populations that can be managed by research teams with limited resources yet large enough to discover genetic factors of moderate to large effects. The CRIB project designed new MA and Andean germplasm panels that are specifically adapted to the climate challenged regions of Central America and Africa. Because of resource constraints for field research in these target regions, the panels were designed to be modest in size (n∼120 lines). Here we report on the development of these moderate-sized panels and the results obtained by combining SNP genotyping data of these panels with those of the MDP and ADP to generate large SNP marker collections for each gene pool. This report also describes the utility of those SNP collections and the new association panels to map genetic factors controlling important production related traits under heat and drought conditions. The utility of multi-trait mixed model (MTMM) GWAS analysis (Korte *et al.* 2012) is demonstrated as a method to identify statistically robust genetic factors in smaller-sized populations. Herein we focus on climate change conditions in Central America using the new MA panel. These populations and SNP data sets are now available to be applied across a broader array of stresses and locations to discover loci and markers that can be applied to other common bean crop improvement efforts.

## Materials and Methods

### Germplasm collection and evaluation panels

Several common bean breeding programs that are partners in this project have been developing cultivars and lines for tolerance to a variety of abiotic stresses. A flexible system for the evaluation of these lines under different abiotic environments is designated here as the Bean Abiotic Stress Evaluation (BASE) approach. Overall, a total of 155 genotypes from the MA gene pool, 147 Andean genotypes, and 5 tepary bean (*Phaseolus acutifolius*) genotypes form the BASE germplasm collection were evaluated in three separate panels (Table S1). The BASE_120 panel consists of 93 genotypes from the MA gene pool, 22 genotypes from the Andean gene pool, and four tepary bean (*Phaseolus acutifolius*) genotypes. The BASE_Meso panel consists of 119 genotypes primarily from Race Mesoamerica within the Middle American gene pool. The BASE_Andean panel contained 124 genotypes. The genotypes forming these panels were obtained from breeding programs at CIAT, Colorado State University, USA; Zamorano University, Honduras; USDA-ARS, Prosser, Washington; USDA-ARS, Puerto Rico; and the University of Puerto Rico. Whereas the BASE_120 and BASE_Meso consist primarily of cultivars and advanced breeding lines, the BASE-Andean contains a large portion of landraces from East Africa.

### Single nucleotide polymorphism data sets

SNP reads from multiple GBS libraries constructed using a two-enzyme protocol [*Mse*I and *Taq*α1; Schröder *et al.* (2016)] of were remapped, and SNPs were called. Those libraries were constructed using genotypes of the MDP, ADP, BASE_120, BASE_Meso, and BASE_Andean populations. MA and Andean SNPs were derived from 482 and 325 genotypes, respectively.

The libraries were sequenced (read length = 230 nt) at the HudsonAlpha Institute for Biotechnology using Illumina HiSeq 2500 Sequencing System. Sequencing barcodes were removed and low-quality sequences were trimmed. Only processed reads with a quality score ≥ 20 and a minimum trimmed length of 180bp were used for mapping. BWA-MEM (Li 2013), and Samtools (Li *et al.* 2009) were used to align the data against reference genome *Phaseolus vulgaris* v2.1, and to index, and sort the aligned reads (https://phytozome.jgi.doe.gov/pz/portal.html#!info?alias=Org_Pvulgaris). The GATK Unified Genotyper v3.3 (McKenna *et al.* 2010) with the minimum confidence threshold of 30 was used to call SNPs. SNPs with <50% missing data were imputed using fastPHASE (Scheet and Stephens 2006).

### Population structure and phylogenic tree analysis

Phylogenetic relatedness of the full set of 807 genotypes, from the panels from which SNP reads were obtained, was investigated by calculating a maximum likelihood (ML) phylogenic tree using the SNPhylo pipeline (Lee *et al.* 2014) with a pairwise LD *r*^2^< 0.1 between consecutive SNPs, and a MAF >0.05. This tree was developed with the 5,637 SNPs shared between the MA and Andean SNP data sets. The tree was bootstrapped with 1,000 iterations using MEGA7 (Kumar *et al.* 2016). In addition, individual trees were constructed for the MA and Andean genotypes separately using the individual population SNPs with the same criteria used to evaluate the full set of genotypes. The shared SNP data set from the phylogenetic analysis of all genotypes was used for a Bayesian population structure analysis with the program STRUCTURE 2.3 (Pritchard *et al.* 2000). An admixture model of independent allele frequencies with 20,000 burn-ins and 10,000 MCMC replication for subpopulation sizes of K = 1 to 10 was implemented (McClean *et al.* 2012). The ΔK statistic parameter (Evanno *et al.* 2005), which calculates the total change in log probability of data between consecutive k values, was used to determine the number of clusters. Final subpopulation graphics were produced by the Distruct 1.1 program. (Rosenberg 2004).

### Phenotypic analysis

Three BASE panels were evaluated in 2014-2016 at the University of Puerto Rico Juana Diaz Experiment Station in Juana Diaz, Puerto Rico (PR) and Nacaome, Honduras (HN). For Juana Diaz, the panels were grown in separate drought and heat experiments, using a lattice design with three replications in 2014, an RCBD design with three replications in 2015, and an RCBD design with six replications under drought and four replications under heat in 2016. All plots were three m in length and row spacing was 0.76 m. The data from Nacaome, HN used a randomized complete block design with three replications of the BASE populations conducted under heat during the dry seasons of 2015 and 2016. The experimental lines were planted on raised 1.5 m wide beds with two rows spaced 0.6 m apart. Row length was 2.5 m. Phenotypic data collected included seed yield (kg ha^-1^), days to flowering (DTF) recorded when 50% of the plants had at least one open blossom, *Macrophomina phaseolina* disease severity scored from 1 to 9 (Abawi *et al.* 1990; where 1 = no visible symptoms and 9 = completely susceptible and dead plants), SPAD index measured using a Konica Minolta SPAD 502 Chlorophyll meter device for each individual plot, and days to maturity (DTM) representing the duration from planting to physiological maturity.

### Genome-wide association studies

GWAS were performed for each trait in each location under different stress conditions using untransformed data. For joint analyses of a phenotype with data from multiple stresses or locations, the data were transformed prior to the GWAS analysis to a standard scale using the statistical Z-transform (the ratio of the deviation of the individual phenotypic value from the population mean to the population standard deviation of the experiment in which the observation was collected). This transformation generates individual phenotypic data that is relative to the overall performance of the population at a specific location under a specific stress condition. In this way, we can pool the Z data across locations or stresses to discover common factors affecting the trait. The association between each quantitative trait and the genome-wide SNPs was analyzed with the GAPIT R package (Lipka *et al.* 2012; R Core Team 2013) as described by Moghaddam *et al.* (2016). A general linear model with fixed effect, and a univariate unified mixed linear model with random effect, and both fixed and random effects were tested for GWAS analysis for each trait. Principal component analysis (PCA) was performed using the Prcom function in R, and relatedness was measured using the EMMA algorithm implemented in GAPIT. Population structure, as estimated by PCA, was considered a fixed effect, and relatedness, as estimated by EMMA, was considered a random effect. Structure was based on the number of PCAs that accounted for 25–50% of the phenotypic variation. The best model was chosen based on the lowest calculated MSD value (Mamidi *et al.* 2011b). Manhattan and QQ plots were generated using SNPs with MAF > 0.05 using mhtplot function from R package gap (Zhao 2007). The significance level was calculated using a Bonferonni test based on the effective number of markers (n = 463) as determine by the simpleM algorithm (Gao *et al.* 2008). The phenotypic variation explained by a significant marker was described as a likelihood-ratio-based R^2^ (R^2^_LR_; Sun *et al.* 2010). This procedure considers the effects of population structure and/or relatedness in the calculation. The analysis was performed with the genABLE R package (Aulchenko *et al.* 2007) to estimate the amount of variation explained by the most significant SNP within a GWAS peak. The relationship between individual SNP genetic effects between two correlated traits (pleiotropy) or environments (genotype-by-environment interaction) was investigated using a multi-trait mixed model (MTMM) as described by Korte *et al.* (2012). Pearson phenotypic, genetic, and environmental correlations and heritabilities were estimated using the MTMM software (Korte *et al.* 2012). For all GWAS analyses, the SNP with the lowest *P*-value was chosen to represent that locus. Only SNPs with minor allele frequency ≥ 0.05 were considered when defining significant loci or regions.

### Candidate Gene Selection

Candidate genes were selected within a ±50 kb interval of the peak SNP within a GWAS peak region. The predicted functional effect of each SNP was obtained from a SNPeff database developed for all SNPs using snpEFF.jar with “build-gff3” (Cingolani *et al.*2012). The snpEff database was used to describe potential effects of SNPs within the ±50kb interval of a peak SNP.

### Data availability

Table S1 contains the list of BASE genotype names. Table S2 contains a summary of MLM GWAS results and reports the peaks for each trait under various environmental conditions.

Table S3 contains SNP distribution across the euchromatic and heterochromatic regions of all chromosomes in two gene pools. File S4 and S5 are text files containing un-imputed HapMap genetic data for Andean and MA genotypes respectively. Supplemental material available at Figshare: https://doi.org/10.25387/g3.7965305.

## Results

### SNP data set development

To maximize the number of SNPs for the haplotype maps, sequencing reads from multiple GBS libraries consisting of individuals with either MA or Andean parentage were pooled. These HapMaps were based on 381,092,199 GBS reads across 469 MA genotypes and 280,085,901 GBS reads across 325 Andean genotypes. These reads averaged 201bp and were mapped against version 2.1 of the *Phaseolus vulgaris* reference genome (phytozome.jgi.doe.gov). Individual MA and Andean haplotype maps (HapMap) were developed after final SNP filtering and imputation. The MA HapMap contained 205,293SNPs, and the Andean HapMap consisted of 260,670 SNPs. The range of SNPs per Mb is consistent across both and heterochromatic of the and Andean SNP data sets (Table S3). For the MA SNP collection, there are 1.79x SNPs in the heterochromatic region compare to the euchromatic region, while that ratio for the Andean SNP collection is 1.51x. The un-imputed HapMap data for each gene pool can be found in S4 and S5 text files. Given the large number of genotypes in each of the two HapMaps, researchers can now design experiments to capture phenotypic data from all or a subset of the genotypes in the HapMap populations and then perform GWAS analyses with a very large SNP dataset to discover important genetic factors controlling traits of interest. The genotypes used to develop the HapMaps represent many of genotypes used for production purposes over the last 50 years in the USA and elsewhere. These HapMaps are a major output from the USAID CRIB project and will be an important genetic resource well beyond the end of the project.

### Population structure and phylogenic analysis results

**Figure 1:**
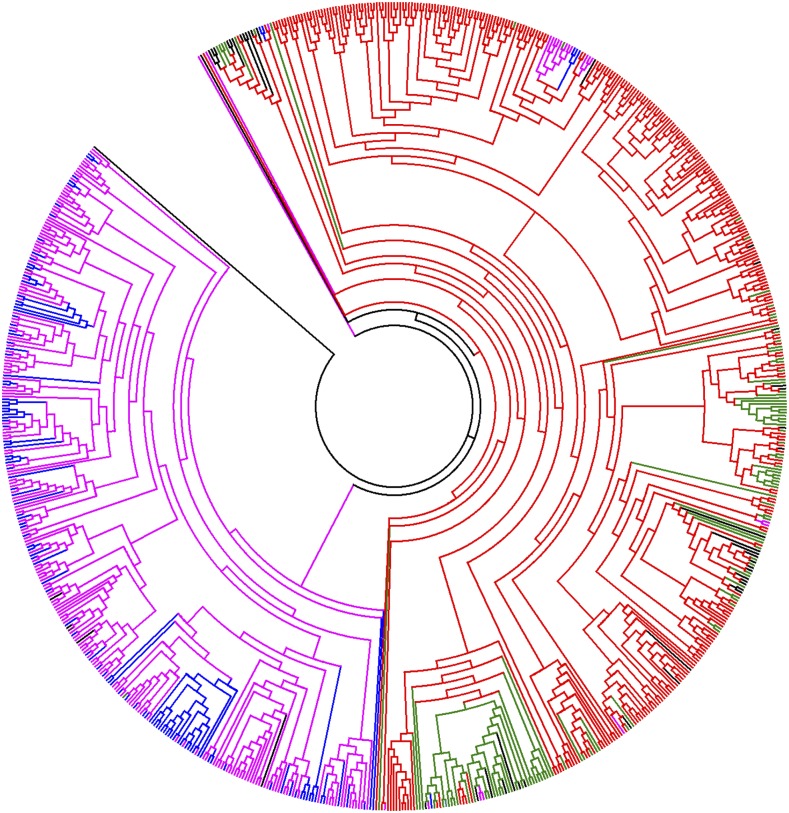
Maximum likelihood phylogenic tree of 769 genotypes from Andean and Middle American gene pools using 5,637 loci with LD < 0.1. Middle American genotypes = red and green; Andean genotypes = purple and blue. BASEMeso = green; BASEAndean = blue.

To develop a full characterization of the genotypes used to generate the SNP datasets, an initial ML tree with 807 MA and Andean common bean genotypes along with a few tepary bean genotypes, was constructed with 5,637 common SNPs with LD < 0.1 (Figure 1). Two distinct clades were observed that separated the MA and Andean genotypes. The BASE_Meso and BASE_Andean are located within their correct gene pools in the tree demonstrating they are appropriate genotypes to study phenotypic responses in the two gene pools. The BASE_Meso genotypes were not distributed across the full spectrum of MA genotypes, rather they clustered in the tree with other known members of race Mesoamerica. This was expected because beans grown in the target Central American region are almost exclusively from race Mesoamerica of the MA gene pool.

**Figure 2:**
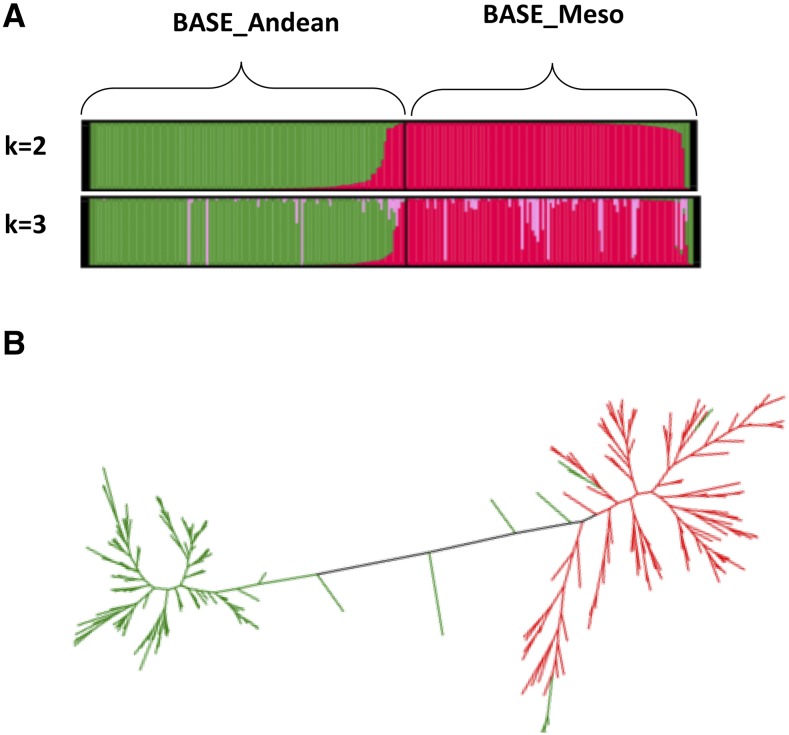
Relationship among the BASE_Meso and BASE_Andean diversity panels. A. STRUCTURE analysis on 242 BASE genotypes with 125k SNPs. k = 2, was optimum number of subpopulations for the BASE populations. B. ML phylogenetic tree of BASE genotypes. Green are the BASE_Meso genotypes and purple and the BASE_Anjdean genotypes

Out of the 102,878 SNPs shared by the two gene pools, a reduced set of 1,882 SNPs with pairwise LD values less than 0.1 were chosen for a Bayesian structure analysis with genotypes used for the BASE populations. The optimum number of subpopulations was k = 2 (Figure 2A) and corresponds to the two BASE panels. The expected heterozygosity between genotypes of the same cluster was 0.29 for BASE_Andean and 0.24 or BASE_Meso. Therefore, the results from STRUCTURE analysis confirmed the two BASE panels represent distinct populations and are appropriate for studies designed to investigate the genetic factors controlling important agronomic traits within each gene pool. This SNP data set will allow researchers to determine whether traits are controlled by genetic factors shared by both gene pools or whether gene pool specific factors are controlling important traits.

The 1,882 SNPs were also used to develop a bifurcated ML phylogenic tree that demonstrated the two populations were clustered into two separate clades (Figure 2B). The BASE_Meso clade branches are more elongated than the shorter branches of the BASE_Andean clade. This supports other observations that the diversity of the Mesoamerican race is greater than that found within Andean genotypes. Eight Andean genotypes (green in Figure 2B**)**, including G13654, G2377, G23829, SAB_6292, SEQ_11, 754_3 and 379_PI_203934, were grouped with BASE_Meso genotypes despite being selected as members of the BASE_Andean panel. Tepary 22 fell in between the two clusters.

### GWAS analyses

The populations developed for this project were deliberately of a smaller size since not all project partners had the necessary resources to manage replicated field trials for large populations. Theory suggests that larger population sizes can uncover either large or small effect size genetic factors while smaller size populations tend to discover only effects of larger size (Korte and Farlow 2013). With these considerations in mind, the three panels of ∼120 individuals were phenotyped in replicated trials in multiple abiotic stress conditions. This phenotype data were then coupled with a robust SNP data set built with reads from a much larger set of individuals from a diverse pool of genotypes that represented the genetic diversity of the two bean gene pools. The phenotypic and genotypic data were then analyzed using single trait mixed linear model (MLM; Yu *et al.* 2006) or multi-trait mixed model (MTMM; Korte *et al.* 2012) GWAS approaches to discover genetic factors associated with several phenotypic traits.

Populations such as those used here that are small and pre-selected for abiotic stress tolerance will also exhibit high LD. Therefore it is important to determine the effective number of genomic regions in that population and using that number when performing a conservative cut-off value test such as Bonferroni. Here we applied the simpleM algorithm (Gao *et al.* 2008) to calculate that number of markers which in turn was used to determine our *P*-value cutoff of -log_10_(*P*) = 4.1.

Pooling data across different experiments to extract factors that affect a phenotype across those experiments normally assumes the population parameters across the two experiments are equal. This will not be the case when the extent of stress at two environments cannot be controlled. So, when data from multiple locations or stresses were merged, Z transformed data were used to provide a common relative estimate of the phenotype (Figure S1). Often the response of two traits, or a single trait scored in two environments are correlated, and the goal of discovering genetic effects associated with these two situations is a goal of quantitative genetics. Recently, multi-trait mixed models (MTMM) statistical methods have been developed to uncover common genetic effects that act in a pleiotropic manner on two correlated traits (Korte *et al.* 2012). It is predicted these effects would be components of a shared functional pathway. The MTMM GWAS methodology has also been applied to the discovery of genetic factors associated with the phenotypic expression of a single phenotype in two different environments. While the full MTMM model uncovers both common and interaction genetic effects, Korte *et al.* (2012) also provide scripts that can partition out these common and interaction effects individually from that full model. Importantly for small population sizes, the power of the MTMM approach is greater than the marginal GWAS tests typical of mixed model statistical methods because of the additional power obtained when data for the two traits (or environments) are considered jointly (Korte *et al.* 2012).

### Mixed-linear model GWAS

#### Yield:

**Figure 3:**
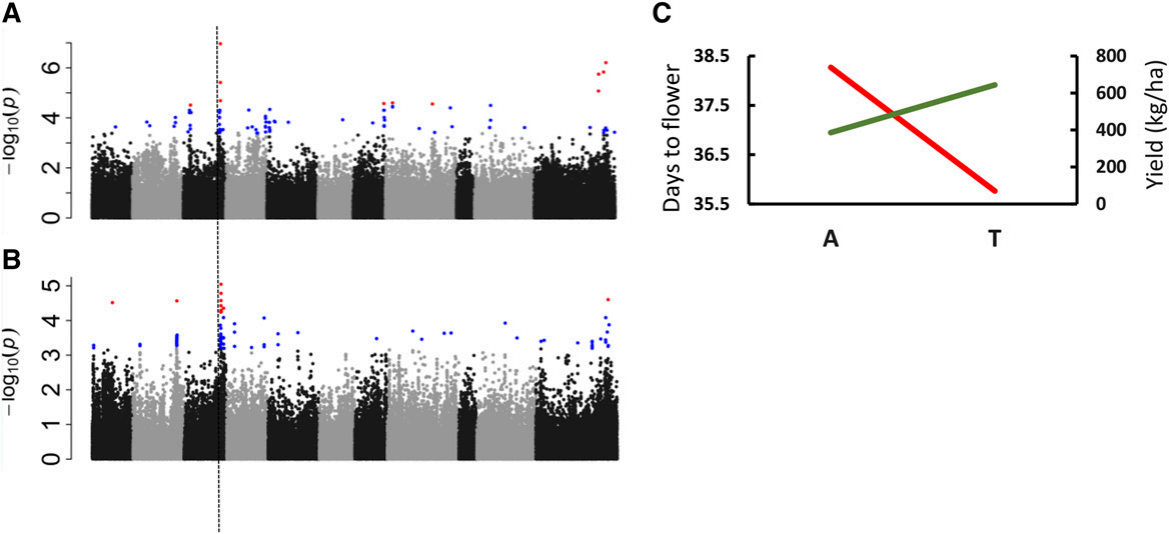
MLM GWAS analysis for BASE panels. A. Yield GWAS results for the panel grown under heat in Honduras and Puerto Rico in 2016. B. Days to flower GWAS results for the panel grown under heat in Honduras and Puerto Rico in 2016. The P value cutoff for the two GWAS, as determined using the Bonferroni test based on the effective number of SNPs was –log10(P)=4.10. C. Allelic performance for SNP S03_ 40504942 for days to flower (red) and yield (green) grown under heat in Honduras and Puerto Rico in 2016.

Yield is the primary target for genetic improvement, and an important genetic goal is to understand the response of yield to a specific stress across locations. As mentioned above, both Nacome, HN and Juana Diaz, PR are high heat stress environments. The effect of heat stress on yield and DTF was assessed on the BASE_Meso population genotypes evaluated in PR in 2015 and 2016, and HN in 2016 (Table S2). Since the three locations were considered different environments with potentially different heat stress conditions, the phenotypic data were transformed using the Z transformation, and the data were combined into a single MLM GWAS analysis (Figure 3A**)**. The peak SNP for yield (Pv03:41,096,424 bp; *P* = 9.05E-8) is located on the distal end of chromosome Pv03 and explains 14% of the variation in yield (Table S2). This peak SNP is located in gene model Phvul.003G187400. Orthologs of this gene model are associated with plants response to heat stress (Li *et al.* 2013). Three other SNPs (Pv08:9,135,122 bp, Pv11: 41,873,950 bp; Pv11: 47,305,350 bp) defined other individual loci, and each accounted for more than 7% of the variation, and along with the peak SNP, these four SNPs collectively accounted for 20.2% of the variation in yield under heat stress (Table S2).

#### DTF:

For DTF at the same location and years, the major QTL peak from the joint MLM analysis was located in the Pv03:40.46-40.50 Mb interval (Figure 3B). The peak SNP was located at 40,504,942 bp (*P =* 9.02E-06) and accounted for 9.9% of the variation. At the proximal end of this interval, gene model Phvul.003G181900 is located. The highest level of expression for this gene was noted in flower buds relative to other developmental and anatomical tissues (https://phytozome.jgi.doe.gov/pz/portal.html#!info?alias=Org_Pvulgaris). Three SNPs are located in this gene model, and these SNPs are in high LD relative to the peak SNP located 36kb distal to Phvul.003G181900. DTF is often a major factor in yield performance. That trend was observed here with a Pearson correlation of *r=*-0.35 between the two traits. To consider that correlation in terms of the strong DTF genetic effect discovered in this analysis, the allelic effects of the major SNP for DTF (Pv03: 40,504,942 bp) on yield was evaluated. Figure 3C shows that selection for the early DTF allele will have a positive effect on yield performance under high heat conditions.

#### Macrophomina phaseolina infection:

**Figure 4:**
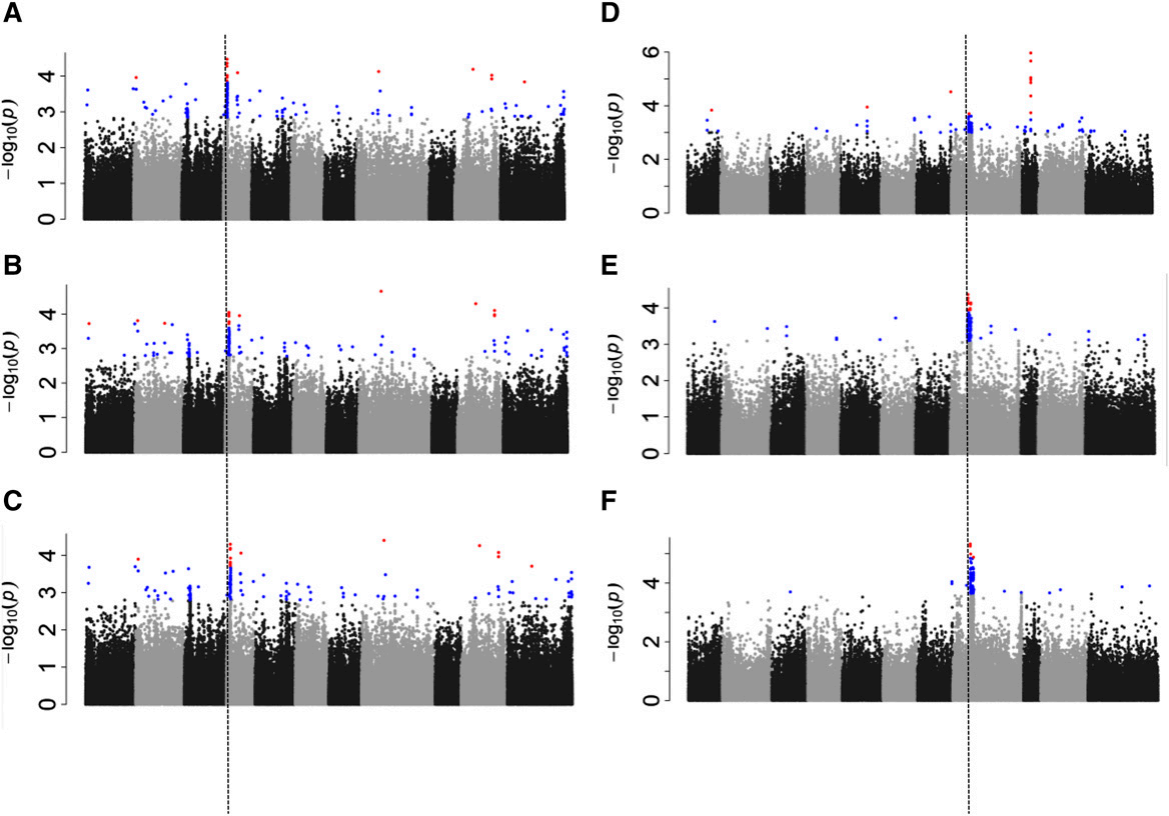
Macrophomina infection rating GWAS for BASE_120 grown under heat in 2014 (A) and drought in 2015 (B) in Puerto Rico. (C) Joint 2014 and 2015 Macrophomina infection rating GWAS analysis for BASE_120 grown under in Puerto Rico. GWAS analysis for SPAD rating for BASE_Meso grown under heat (D) and drought (E) in Puerto Rico in separate trials in 2016. (F Joint heat and drought SPAD reading GWAS analysis.

Field *M. phaseolina* infection data were collected on the BASE_120 population grown in PR in 2014 under heat stress and in 2015 under drought stress. The data were standardized using the Z transformation. The individual GWAS results for the two years were consistent, and the same major locus was discovered at Pv04/4.64-4.84 Mb (Figure 4A, 4B). The peak SNP in each analysis was located at Pv04:4,665,828 bp and accounted for 8.9 and 7.8% of the variation, respectively, for the heat and drought trials. When the data from the two years were combined, the joint GWAS analysis with data from the two stresses also discovered the same major QTL interval and peak SNP (*P* = 5.04E-5; Figure 4C). Consistent with the individual trials, the peak SNP accounted for 8.4% of the variation (Table S2**)**. Additional SNPs were located at Pv04:25,282,114 bp (*P* = 5.04E-5) and Pv10: 32,029,428 bp (*P* = 5.04E-5) in the combined analysis. Collectively, these three SNPs accounted for 17.6% of the observed variation. The A allele at the peak SNP was associated with lower disease incidence in the two trials. This SNP peak is located in one of the two major clusters of malectin/receptor-like kinase genes in the common bean genome.

#### SPAD:

SPAD readings are a general indicator of greenness of the plant. Given proper flowering conditions, this trait can be an indicator of yield potential. A MLM GWAS analysis was performed in separate heat and drought environments in PR in 2016 using the BASE_Meso population. Again, because the trials were under two stress conditions, Z transform data were evaluated. This trait appeared to be under different controls under the two conditions. The major SNP peak under heat (Figure 4D**)** was located at Pv09:17,981,113 (*P* = 1.08E-6) and accounted for 12.7% of the phenotypic variation (Table S2). This peak QTL region is located in a cluster of chitinase genes. For drought, the strongest SNP peak was located in the heterochromatic region between Pv08 18.4-21.5 Mb (Figure 4E**)** and accounted for 7.0% of the variation. This interval was also detected in the combined analysis (Figure 4F**).**

### Multi-trait mixed-linear model GWAS

**Figure 5:**
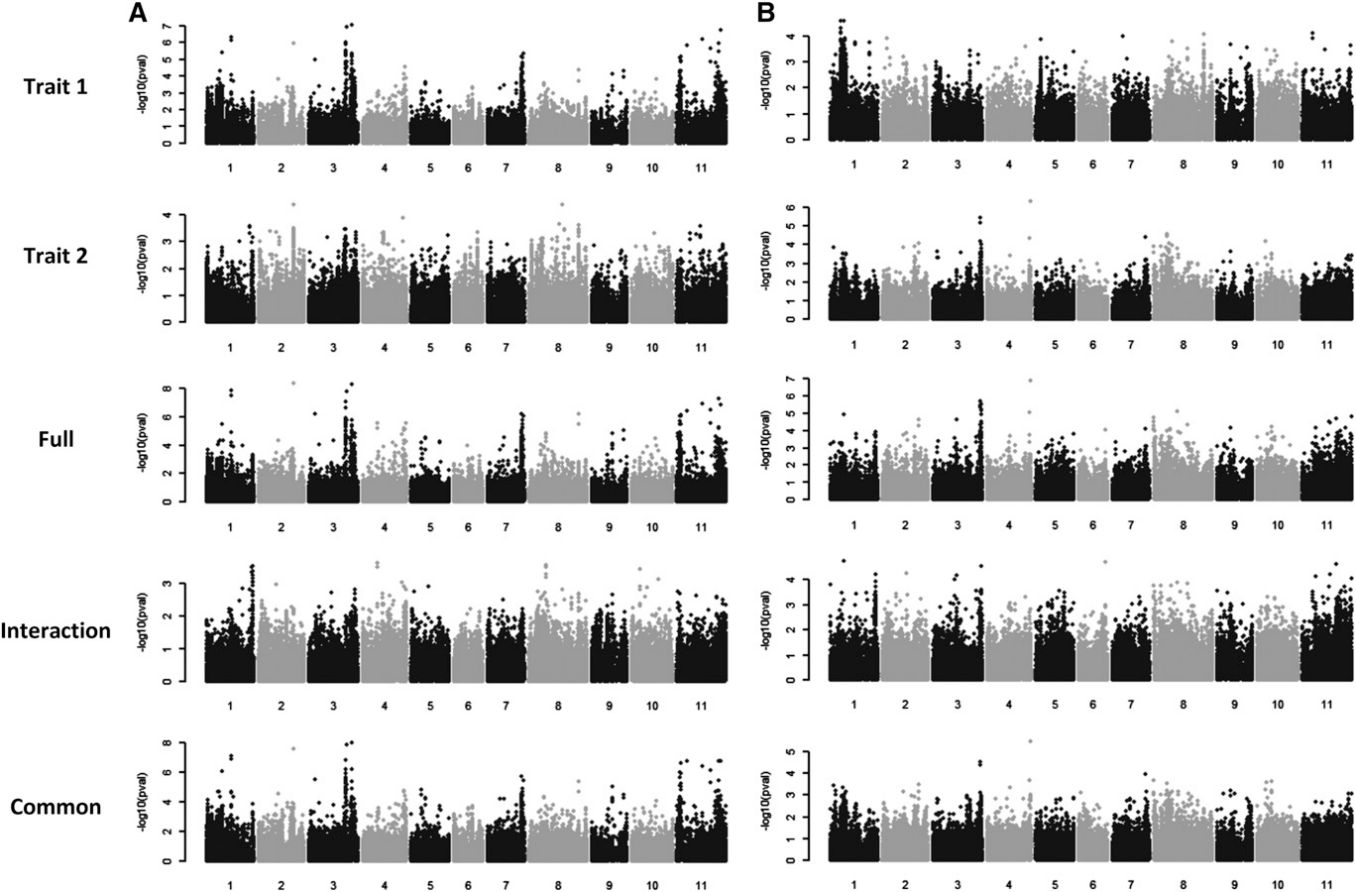
Multi-trait mixed model GWAS. A. Days to flower in Honduras, (Trait 1) and Puerto Rico (Trait 2) grown in 2016. B. Days to flower (Trait 1) and days to maturity (Trait 2) grown in Puerto Rico in 2016. The full model identifies those SNPs with either an interaction or common effect. The interaction model identifies SNPs that act differentially for the two traits or locations. The common model identifies SNPs that act in the same direction for the two traits or locations. The P value cutoff for the two GWAS, as determined using the Bonferroni test based on the effective number of SNPs was –log10(P)=4.10.

The first MTMM analysis evaluated DTF measured in HN and PR under heat stress conditions in 2016 (Figure 5A). The full MTMM analyses showed that genetic correlations were significant (*r* = 0.96), while the environmental correlations were not (Table 1). This result suggests that genetic factors that are common or show an interaction effect of significance between the two heat stress environments may be discovered. Eleven different QTL regions were discovered with a MAF > 0.05 that passed the Bonferroni cut-off in at least one of the analyses (Table 2). The power of the MTMM approach is demonstrated here by the observation that no genetic factor passed the strict Bonferroni cut-off in the marginal test in PR (Table 2). Yet, when the data from the two locations were considered jointly, the full model revealed multiple genetic factors affecting DTF in the two locations that passed the conservative Bonferroni significance cutoff. Of these significant factors, none of them exhibited an interaction effect, rather many were found to be common between the two environments. This is encouraging for marker assisted breeding because only a single or a few markers may be needed for selection for days to flower in these two heat stress environments. Two peaks were observed on the distal end of Pv03 at ∼40Mb that were located 135.2 kb apart. On Pv11, significant peaks were observed at 4.0 Mb and 45.3 Mb. These two are significant common factors and had the same positive effect at both locations (Figure 5A). Within these regions, significant SNPs were located in three candidate gene models (Phvul.003G179500, Phvul.003G187400, and Phvul.011G159200). Candidate genes that are orthologs of genes previously found to be involved in flowering and abiotic stress response are located within 50kb of the significant peaks (Phvul.003G239000, Phvul.011G045000).

**Table 1:** Pearson phenotypic, genetic and environmental correlations and joint heritability estimates for environmental DTF HN 2016 & DF PR 2016 and DTF PR 2016 & DTM PR 2016 combinations

		Genetic			Environmental			
Traits	Pearson Corr.	Corr.	St. error	*P*-value	Corr.	St. error	*P*-value	Joint heritability*^a^*
DTF HN 2016 & DF PR 2016	0.55	0.96	0.07	0.00	−0.18	0.33	0.51	0.71/0.71
DTF PR 2016 & DTM PR 2016	0.68	0.90	0.07	0.00	−0.40	0.87	0.53	0.89/0.75

a Joint heritability estimates for the two traits are separated by a “/”.

**Table 2:** Significant associations for days to flower measured in heat conditions in Nacaome, Hondouras (HN) and Juana Dias, PR (PR) on the BASE_Meso panel in 2016. The MTMM statistical method and scripts (Korte *et al.* 2012) were used to calculate the marginal associations in HN (DTF HN) and PR (DTF PR), the full mixed trait model (full), and the models that tested for interaction and common effects for the two traits. SNPs that passed the Bonferonni test based on the effective number of markers (n = 463; Gao *et al.* 2008; -log_10_ ≥ 4.10) for any of these analyses are shown in bold font. Minor-allele frequency ≥ 0.05

				MTMM test [-log_10_(P)]				
SNP	Chr	Position	MAF	DF HN	DF PR	Full	Interaction	Common
S01_27685396	1	27,685,396	0.094	**6.33**	0.36	**7.83**	2.19	**7.07**
S01_27685497	1	27,685,497	0.094	**6.16**	0.49	**7.48**	1.98	**6.90**
S02_38071921	2	38,071,921	0.052	**5.97**	0.85	**8.32**	2.17	**7.60**
S03_40276852	3	40,276,852	0.175	**6.02**	1.49	**7.09**	1.66	**6.78**
S03_40412039	3	40,412,039	0.189	**5.89**	1.58	**6.63**	1.81	**6.18**
S03_41096424	3	41,096,424	0.052	**6.96**	1.55	**7.78**	1.28	**7.84**
S03_47356534	3	47,356,534	0.113	**7.07**	1.65	**8.29**	1.72	**7.98**
S11_4021661	11	4,021,661	0.085	**5.16**	1.75	6.02	0.60	**6.57**
S11_10662631	11	10,662,631	0.090	**5.87**	1.26	6.39	0.91	**6.72**
S11_10662636	11	10,662,636	0.090	**5.87**	1.26	6.39	0.91	**6.72**
S11_10662653	11	10,662,653	0.090	**5.87**	1.26	6.39	0.91	**6.72**
S11_10662667	11	10,662,667	0.090	**5.87**	1.26	6.39	0.91	**6.72**
S11_10662679	11	10,662,679	0.090	**5.87**	1.26	6.39	0.91	**6.72**
S11_27278152	11	27,278,152	0.094	**6.22**	0.36	**6.89**	1.87	**6.39**
S11_27278162	11	27,278,162	0.094	**6.22**	0.36	**6.89**	1.87	**6.39**
S11_27278186	11	27,278,186	0.094	**6.22**	0.36	**6.89**	1.87	**6.39**
S11_36223457	11	36,223,457	0.071	**5.67**	0.34	**6.45**	1.66	**6.13**
S11_45290191	11	45,290,191	0.094	**5.97**	1.02	**7.28**	1.91	**6.77**
S11_47305350	11	47,305,350	0.057	**6.73**	1.88	**6.82**	1.39	**6.76**

The objective of the second MTMM analysis was to discover genetic factors with pleiotropic effects for both DTF and DTM under heat stress (Figure 5B). The experiment with the BASE_Meso in PR in 2016 population found 1) a significant genetic correlation between the two traits, 2) the environmental effects were not significant, and 3) both traits were heritable. Marginal effects of a magnitude of –log10(*P*) > 5.0 were only observed for DTM. For the full MTMM model, eight genomic regions passed the –log10(*P*) > 5.0 threshold. Only one region, Pv11:47.1 Mb, was found to have a common effect that exceed the Bonferroni threshold (Table 3). 20kb upstream of the significant SNP was a gene model (Phvul.004G166400) that is homologous to a tomato gene that affects flowering and other growth functions (Bassa *et al.* 2013). None of the effects acted differentially between DTF and DTM. Therefore, selection on these markers can have positive effects in the same direction for both traits.

**Table 3:** Significant associations for days to flower (DTF) and days to maturity (DTM) measured in Nacaome, Juana Dias, PR on the BASE_Meso panel. The MTMM statistical method and scripts (Korte *et al.* 2012) were used to calculate the marginal associations for DTF and DTM, the full mixed trait model (full), and the models that tested for interaction and common effects for the two traits. SNPs that passed the Bonferonni test based on the effective number of markers (n = 463; Gao *et al.* 2008; -log_10_ ≥ 4.10) are shown in bold font and underlined. Minor-allele frequency ≥ 0.05

				MTMM test [-log_10_(P)]				
SNP	Chr	Position	MAF	DTF	DTM	Full	Interaction	Common
S03_51487987	3	51,487,987	0.08	0.76	**4.20**	**5.32**	3.54	3.13
S03_51737958	3	51,737,958	0.13	2.01	**5.43**	**5.69**	2.52	**4.52**
S03_51737987	3	51,737,987	0.11	2.10	**5.19**	**5.46**	2.39	**4.41**
S03_51773012	3	51,773,012	0.11	2.10	**5.19**	**5.46**	2.39	**4.41**
S03_51773026	3	51,773,026	0.11	2.10	**5.19**	**5.46**	2.39	**4.41**
S03_51977930	3	51,977,930	0.08	0.63	4.03	**5.23**	3.60	2.96
S03_51978029	3	51,978,029	0.08	0.63	4.03	**5.23**	3.60	2.96
S03_51978076	3	51,978,076	0.08	0.63	4.03	**5.23**	3.60	2.96
S03_52011377	3	52,011,377	0.08	0.63	4.03	**5.23**	3.60	2.96
S03_52321115	3	52,321,115	0.09	0.57	4.01	**5.14**	3.57	2.90
S03_52607649	3	52,607,649	0.08	0.09	3.58	**5.54**	**4.55**	2.32
S04_46335883	4	46,335,883	0.40	1.11	**4.33**	**5.07**	2.72	3.68
S04_47068592	4	47,068,592	0.28	2.13	**6.35**	**6.86**	2.81	**5.47**
S08_24957350	8	24,957,350	0.20	0.08	3.82	**5.13**	3.89	2.56

## Discussion

As a species, *P. vulgaris* is somewhat unique in that the wild ancestor split into two wild gene pools, the MA and Andean, ∼100k years ago (Gepts *et al.* 1986; Mamidi *et al.* 2013; Schmutz *et al.* 2014). Only recently did these gene pools undergo independent domestications about ∼7k years ago (Mamidi *et al.* 2011a; Schmutz *et al.* 2014) in distinct locations to form two distinct domesticated clades. Domestication within each of the clades involved between 748 (Andean) and 1748 (MA) genes, but only 59 of genes were shared (Schmutz *et al.* 2014). And when the same gene is involved in the domestication, recent research has shown convergent evolution produced unique alleles in each gene pool that were associated with the domesticated phenotype (Kwak *et al.* 2012; McClean *et al.* 2018). GWAS experiments are also revealing that adaptation to environmental stress conditions evolved differentially in the two gene pools as exemplified by the discovery that distinct genetic factors are associated with the response to flooding in the two gene pools (Soltani *et al.* 2017, 2018). These independent evolutionary paths have also affected marker development and deployment, most notably for disease resistance markers where quite often a specific marker is only diagnostic in a one gene pool (Miklas *et al.* 1993, 1996) while being monomorphic in the other pool regardless of whether the genotype is resistant or susceptible. This is the result of the strong population structure and distinct linkage disequilibrium (LD) arrangements in the two gene pools.

From the perspective of developing association panels, the unique LD structure within the two bean gene pools and the repeated observation that phenotypes are often controlled by different genetic factors in the two pools makes it imperative that genetic experiments of bean be practiced within distinct MA and Andean panels. The first GWAS panels developed for common bean, the MA (Mafi Moghaddam *et al.* 2016) and Andean (Cichy *et al.* 2015a) Diversity Panels, where used to survey phenotypic variation in U.S. commercial and African landrace germplasm, respectively. In contrast, here the BASE_120 and the BASE_Meso panels were developed for the purposes of mapping genetic factors in germplasm important for Central America and Caribbean production regions. The selection of genotypes was successful as evidenced by the phylogenetic analysis which shows that BASE_Meso genotypes cluster with other genotypes from race Mesoamerican, the predominant race grown in these regions. Because the BASE_Andean panel was developed for research in African countries, a subset from the Andean Diversity Panel was chosen. As expected, these genotypes clustered with other germplasm from the Andean gene pool.

Maximizing the number of SNPs within any collection of genotypes will increase the likelihood of finding associations with a target phenotype in the full collection or a subset of the genotypes. To leverage the full set of GBS projects in common bean, all GBS reads from libraries based on the two-enzyme (Schröder *et al.* 2016) protocol were pooled, and new SNP calls made. This increased the number of SNPs for MA genotypes from ∼160k to ∼205k. Previously, the ADP had only been genotyped with the ∼6k SNPs from the BARCBean 6K_3 BeadChip (Song *et al.* 2015). There are now ∼260k SNPs available for a large collection of Andean genotypes. The density of SNPs is essentially equal across the full genome of the two gene pools with an enrichment of SNPs in the heterochromatic regions. This is actually a positive feature because it will facilitate the mapping of phenotypes whose genetic control is located in these low recombination regions of the genome. These SNP data sets can also serve as a base to build much larger SNP sets such as those developed for maize (Glaubitz *et al.* 2014).

One persistent challenge when searching for important genetic factors related to a trait of interest is performance across locations. These locations can represent similar environments such as regional crop production sites that experience somewhat similar weather patterns or diverse sites that cross national or continental boundaries. The critical step is placing the phenotypic data on a standard scale. The standard score (or Z transformation) is ideal for this purpose because phenotypic values are scaled relative to the variation at the location. By pooling standard score data across locations, a full data set is utilized and a more accurate measure of the effect of specific genetic physical positions can be assessed. The usefulness of this approach was demonstrated when we compared standard DTF data pooled across locations and Macrophomina data pooled across stresses. For both traits, the pooled data identified the same significant peak SNP regions that were observed in the individual analyses with the untransformed data. MTMM methods are another way of maximizing the data that is collected (Korte *et al.* 2012). These analyses provide a statistical framework for multiple tests that can reveal common genetic effects that affect two traits or one trait in two environments. In addition, MTMM testing can uncover interaction genetic effects that act in the opposite direction between two traits or for a single trait in two environments. In the latter case, this interaction reflects the genotype x environment interaction effect that is important in the context of breeding for multiple environments. The combination of data for two traits or environments can lead to the discovery of stronger effects than those discovered using a single marginal analysis (Korte *et al.* 2012). This is a direct advantage for a project with more limited resources because statistically sound results can reveal important genetic relationships that would not have been detected with a MLM analysis with smaller panel sizes. We tested that premise on two data sets. The MTMM analysis of DTF data from the BASE_Meso population grown in HN and PR under heat in 2016 showed the full joint analysis out-performed individual marginal analyses (Figure S2). The full model also out-performed the individual marginal analyses when DTF and DTM data were considered jointly.

In one case, it is useful when comparing two locations and searching for SNPs associated with differential (or GxE) effects or SNPs that condition a common response in both locations. This begins with a determination of the genetic correlation of the response in the two locations. For DTF, this correlation was high (r = 0.96) and very significant (Table 1), and without environmental effects. This suggested that common genetic effects were controlling the phenotypic response in the two environments. From a plant breeding perspective, the development of molecular markers that are functional across locations should be possible. MTMM is also useful to determine SNP effects associated with more than one trait. An example is DTF and DTM, two traits often found to be correlated. These two traits had a very high and significant genetic correlation (r = 0.90) that lacked an environmental correlation. In this example, the MTMM full model was significant for 14 common effect SNP loci.

Candidate genes were selected from an interval that ranged from 50kb upstream to 50kb downstream of the peak SNP using the common bean v2.1 gene models (https://phytozome.jgi.doe.gov/). The peak QTL region for DTF in the joint MLM analysis under heat stress in HN and PR is located on Pv03 at 40.48-40.50 Mb. At this position, three SNPs are located in Phvul.003G181900, an ortholog of the Arabidopsis *BIM1* gene. In Arabidopsis, BIM1 functions in the brassinosteroid pathway to regulate flowering through its interaction with SPL8 to promote anthesis (Xing *et al.* 2013). Previous research demonstrated that heat stress in common bean causes indeshiscent anthers and abnormal pollen in heat sensitive genotypes (Porch and Jahn 2001). These observations support a role of the bean *BIM1* ortholog as a strong candidate gene for regulating flowering under heat stress. The peak common effect for DTF in the MTMM analysis of flowering under heat stress in HN and PR was also on Pv03 and mapped 40kb (and one gene away) from Phvul.003G239000 at Pv03:47.36 Mb. This gene is the bean homolog of Arabidopsis *HOS15*, a gene associated with histone deacetylation and epigenetic control of flowering (Zhu *et al.* 2008). The primary role of *HOS15* is the regulation of flowering under cold stress. The major genetic association of *HOS15* with flowering under heat stress might suggest the gene may act as control factor under multiple temperature stresses.

The peak SNP discovered in a joint MLM analysis for yield over years in the HN and PR heat stress environments is located in gene model Phvul.003G187400. This SNP was annotated as a missense variant. This model contains a DUF538 domain that is a key signature of the DUF538 superfamily whose members are well-known stress-related proteins in plants (Gholizadeh 2016). A previous study on switchgrass showed that a DUF538 domain protein was significantly up-regulated in leaves under high heat conditions while expression was very low under normal conditions (Li *et al.*, 2013). DUF538 proteins are putative chlorophyll hydrolyzing enzymes that function in the ROS detoxification system when the plant is exposed to heat and drought stress (Gholizadeh *et al.* 2015 and Li *et al.* 2013), and ROS detoxification enhances heat and drought stress tolerance (You and Chan 2015). Because the exact function of DUF538 proteins is yet unknown, the genetic association of this gene as a yield factor under heat stress may provide a link between cytosolic protection (Gholizadeh 2016) and yield performance. This important molecular link to yield is suggested by the fact that the SNP in this gene accounts for 13.7% of the variation in yield.

Leaf senescence and the associated loss of greening is a result of multiple stresses on the plant including excessive heat (Lim *et al.* 2007). The SPAD rating under heat stress is one indicator of variation in the response to heat stress. SPAD-related QTL can point to important genetic factors associated with one aspect of the heat stress response. The peak MLM QTL for SPAD rating under heat stress is located at Pv09:17.99 Mb, and this QTL is located within a cluster of basic chitinase genes. The previous observation that chitinase genes are involved in both leaf development and senescence (Quirino *et al.* 2000) points to a gene in this cluster as strong candidate gene for the whole plant response under heat stress.

The two peak SNPs for response to *M. phaseolina* in the MLM analysis under heat and drought stress are located at positions Pv04:4,639,929 bp and Pv04: 4,639,994. These SNPs are located within a cluster of seven Malectin/receptor-like protein kinase genes. Another large cluster of Malectin/receptor-like protein kinase genes is located on Pv08. This Pv08 cluster contains multiple paralogs of the bean COK-4 gene, and one of the bean paralogs was recently shown to rescue Arabidopsis mutant *FER* lines susceptible to *Pseudomonas syringae* (Azevedo *et al.* 2018). Receptor protein kinase genes are one component of the plant immune signaling system (Tena *et al.* 2011), and in beans and other species they are involved in the immune signaling pathway that is initiated after pathogen invasion (Azevedo *et al.* 2018; Stegmann *et al.* 2017; Minkoff *et al.* 2017; Masachis *et al.* 2016 and Kessler *et al.* 2010). The regulation depends on the perception of a hormone peptide called RALF. The malectin/receptor kinase/RALF complex has a negative effect on the plant immune system by preventing the modulation of FLS2-BAK1 (FLAGELLIN-SENSING2/ BRASSINOSTEROID INSENSITIVE 1–associated kinase) complex mediated by FER. FLS2-BAK1 has a positive effect on plant immune system after pathogen invasion by initiating an immune signaling at the plant cell membrane. Therefore, mutations affecting the formation of malectin/receptor kinase/RALF complex will lead to disease following pathogen invasion (Azevedo *et al.* 2018; Stegmann *et al.* 2017). The discovery that a common bean malectin/receptor-like protein kinase homolog can act as a disease resistance gene supports the hypothesis that a member of the Pv04 cluster can be a strong candidate to provide *M. phaseolina* resistance.

In general, these GWAS results demonstrate that significant factors with relative high effects can be discovered using moderate size populations along with high-density SNP data sets using single and multi-trait analyses. These analyses will discover polymorphisms within genes with strong candidate credentials, and these SNP polymorphisms can be used as selection tools for traits important for high crop productivity. This makes it now possible for groups of bean researchers with modest resources to use the panels and SNP data sets developed here to search for genetic factors and polymorphisms that would be useful for improvement in their breeding programs.
